# Recovery of culturable *Streptococcus pyogenes* from swabs stored at different temperatures

**DOI:** 10.1111/1758-2229.70036

**Published:** 2024-11-08

**Authors:** Kate Summer, Steven Y. C. Tong, Jonathan R. Carapetis, Asha C. Bowen

**Affiliations:** ^1^ Wesfarmers Centre for Vaccines and Infectious Diseases, Telethon Kids Institute University of Western Australia Nedlands Western Australia Australia; ^2^ Victorian Infectious Disease Service, The Peter Doherty Institute for Infection and Immunity, The Royal Melbourne Hospital Melbourne Victoria Australia; ^3^ Department of Infectious Diseases, The Peter Doherty Institute for Infection and Immunity The University of Melbourne Melbourne Victoria Australia; ^4^ Department of Infectious Diseases Perth Children's Hospital Nedlands Western Australia Australia

## Abstract

Improving our understanding of superficial *Streptococcus pyogenes* (Strep A) carriage and transmission necessitates robust sampling methods. Here, we compared the effect of storing swab samples in fridge (+4°C) and freezer (−20°C) conditions on the recovery of laboratory‐cultured *S. pyogenes*. *Streptococcus pyogenes* colony‐forming units progressively declined at +4°C, but not at −20°C, over 2 weeks. Results demonstrate that freezing is preferable over refrigeration for storage and transport of skin and throat swabs to ensure that culturing accurately reflects the true results of sampling. This is particularly important in remote community research and practice when immediate incubation is not possible or batch processing is most practical, increasing the elapsed time between collection and laboratory analysis. The study suggests that temperature negatively affects *S. pyogenes* viability and provides a method to further investigate the role of other environmental factors affecting *S. pyogenes* transmission.

## Graphical abstract

In this study, we compared the effect of storing *Streptococcus pyogenes* (Strep A) ATCC 19615 swab samples in fridge (+4°C) and freezer (−20°C) conditions. Over 2 weeks, colony counts progressively declined at +4°C, but not at −20°C. These results indicate that swabs should be stored frozen rather than refrigerated to ensure that culturing accurately reflects the sample. Further research using similar methods is needed to investigate the broad range of environmental factors affecting the viability of *S. pyogenes.*

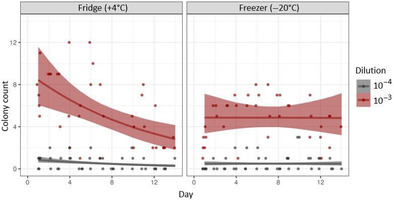

## INTRODUCTION

*Streptococcus pyogenes* (Group A Streptococcus [GAS] or Strep A) infections and complications contribute to an inequitable and preventable burden of disease. They disproportionately affect people living in disadvantaged and remote communities as a result of inadequate housing conditions, reduced access to healthcare and long‐term lack of control over those determinants (Ghamari et al., 2022; Katzenellenbogen et al., 2017; Ralph et al., 2022). Persistent and recurrent superficial *S. pyogenes* infections manifesting as impetigo or pharyngitis (Bowen et al., 2014; McDonald et al., 2007; Oliver et al., 2018) can lead to the development of more serious invasive disease and post‐infection sequelae, including acute rheumatic fever (ARF) and rheumatic heart disease (RHD) (Carapetis et al., 2016; Wiegele et al., 2023). Whilst the pathophysiology of *S. pyogenes* has been extensively studied (Brouwer et al., 2023), its survival/viability in the inanimate environment is less well known (Wagenvoort et al., 2005).

There is a need to better understand the carriage and modes of transmission of *S. pyogenes* and evaluate the efficacy of interventions (Lacey et al., 2023; Wyber et al., 2020). Sampling strategies, which usually involve swabbing, must therefore be robust and reliable. Since environmental factors (e.g., temperature, light, humidity) may affect cell viability (Ross et al., 1982), storage conditions and elapsed time before samples reach the laboratory are important considerations, particularly in remote community‐based research and clinical practice. At present, there are substantial gaps in knowledge regarding the effects of environmental factors on *S. pyogenes* viability, and methods to readily make these assessments are needed.

Here, we assessed the recovery of culturable *S. pyogenes* from inoculated swabs stored either in fridge (+4°C) or freezer (−20°C) conditions to (a) guide the most appropriate storage time and transportation method available to store samples prior to transport from remote communities to an urban laboratory, and (b) contribute to understanding of the effect of temperature on *S. pyogenes* viability and transmission. The method may also be applied for further investigation into the effect of different environmental conditions that may impact the viability of *S. pyogenes*.

## EXPERIMENTAL PROCEDURES

*S. pyogenes* ATCC 19615, originally isolated from a child with pharyngitis, was selected for this study. This non‐motile, Group A β‐hemolytic strain is widely used for laboratory testing and quality control as two emm (M protein) genes are encoded, serotypes 5 and 49 (Minogue et al., 2014), which are associated with throat and skin infections, respectively (Cunningham, 2000). To prepare the experimental suspension, the cryopreserved stock was thawed then streaked onto horse‐blood agar (HBA) and incubated for 18–24 h at 37°C with 5% CO_2_. A suspension was prepared to a concentration of 0.5 McFarland in 4 mL 0.9% normal saline by colourimetry (abs 0.08–0.1 at 625 nm) and vortexed at 1200 rpm to mix. This is equal to approximately 1 × 10^8^ colony‐forming units [CFU]/mL.

Sterile cotton‐tipped swab sticks were soaked in the suspension, placed in individual BD Microtainer® vials containing 1 mL skim milk tryptone glucose glycerol (SMGG) broth transport medium, cut with sterilized scissors, and capped. A total of 56 swab‐containing vials were prepared: 28 were stored in a refrigerator at +4°C and 28 were stored in a freezer at −20°C. Two samples per treatment were assessed daily over the following 14 days. Samples were thawed, vortexed and then diluted 1:10 in 0.9% normal saline before three serial dilutions were carried out: 1:100 (10^−2^), 1:1000 (10^−3^), and 1:10,000 (10^−4^). Samples were capped and vortexed between dilutions. Three 10 μL samples from each dilution were dropped in quadrants of HBA plates, which were then incubated at 37°C in 5% CO_2_. After 24 h, the number of colonies were counted and recorded.

Raw colony count data were fit to a general linear model using the package ‘brms’ in R (v. 4.4.0) (Bürkner, 2017). Data were modelled as a function of count and day, their interaction, a random sample effect, and the dilution as an offset. Counts with a standard deviation greater than 3 based on triplicate drops on each plate were considered outliers. Significance (*p* < 0.05) was assessed based on whether zero was contained within the 95% credible intervals (CI's).

## RESULTS

**FIGURE 1:**
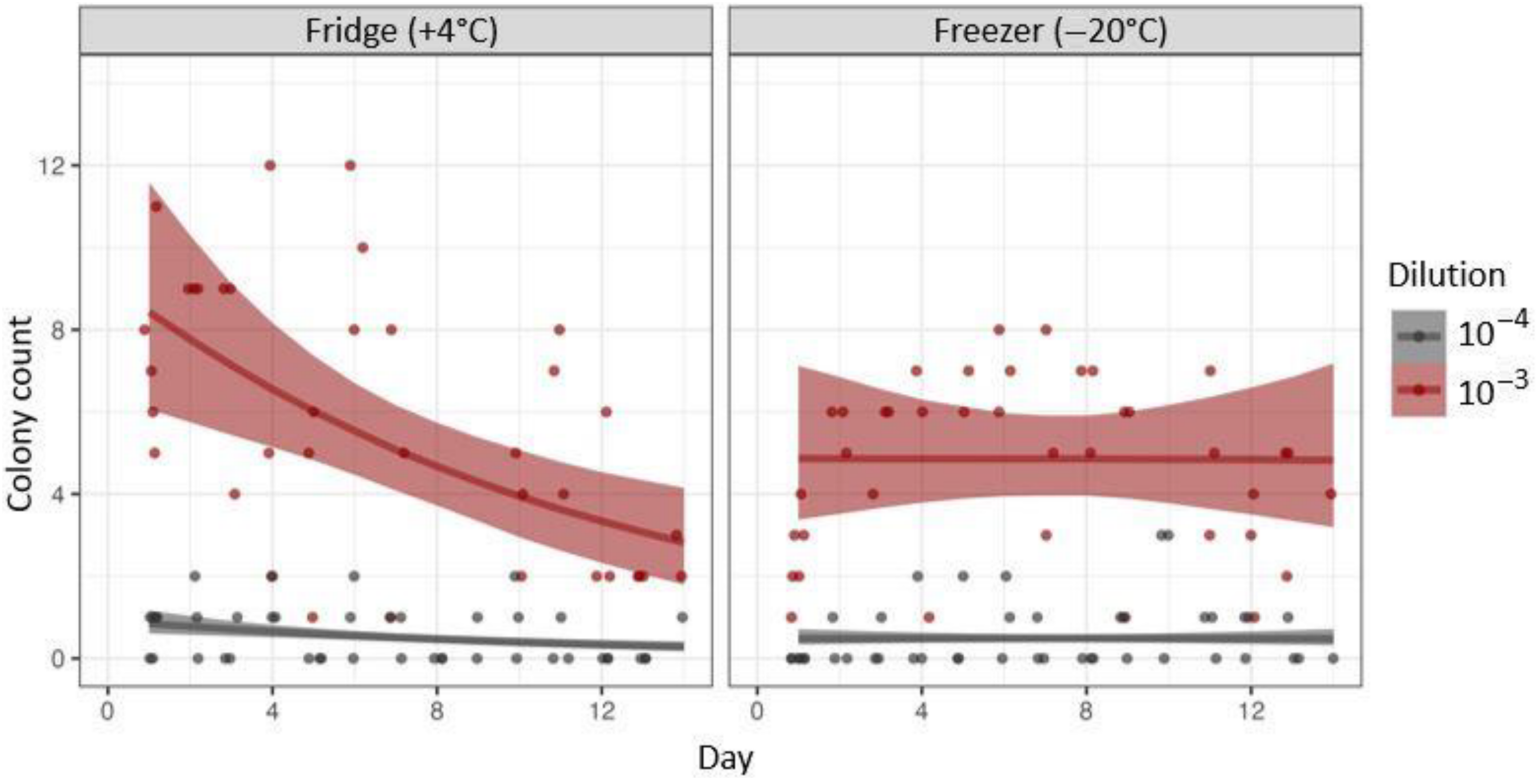
Viable *Streptococcus pyogenes* colony counts from swabs stored in fridge (+4°C) and freezer (−20°C) conditions determined daily over 14 days. There was a reduction in viable *S. pyogenes* colony counts with increasing days of refrigeration, but not when samples were stored frozen. Colony count data were useful when samples were diluted to 10^−3^, but not at higher (10^−4^, lower colony counts) or lower dilutions (10^−2^, formed lawn).

*S. pyogenes* CFU counts declined significantly with an increasing number of days when swab samples were stored in the fridge (+4°C) (Figure 1). There was no effect of day when samples were stored in the freezer (−20°C) (Figure 1). Between days 1 and 4, the average recovery of culturable *S. pyogenes* from fridge storage (6.3 CFU) was higher than from freezer (4.4 CFU), but variation was greater (1.5 and 1.0 standard deviations, respectively) (Figure 1). Colony count data were most useful at the 10^‐3^ dilution (Figure 1).

## DISCUSSION

Our findings show that the recovery of culturable *S. pyogenes* progressively declines at +4°C, but not at −20°C, over a period of 2 weeks (Figure 1). Consequently, refrigeration may be suitable in the short term (i.e., 0–4 days), but freezing is preferable for longer‐term storage and transport of skin/throat swabs between collection and laboratory analysis to ensure that culturing best reflects the initial sampling inoculum. Using the standard suspension provides a consistent and conservative estimate of the temporal effects of temperature since initial inoculum concentrations from clinical samples are typically higher, and vary substantially by sample location (e.g., purulent/crusting skin sores, throat) and swabbing technique. It should be noted that, whilst the ATCC 19615 strain is considered broadly representative, the results from this study cannot be inferred to all strains of *S. pyogenes*, and further work is needed using clinical isolates.

The results of this study are in alignment with Wagenvoort et al., (2005) who found that exposure to ambient temperature and humidity (and subsequent desiccation) caused a rapid decline in *S. pyogenes* viability, suggesting that prolonged survival outside of the human host is not an important factor in the dynamics of transmission for this pathogen (Wagenvoort et al., 2005). Notwithstanding, the role of body fluids and fomites as factors possibly related to viability and transmission must be considered. The interaction between nutritional factors, metabolic products, humidity and temperature is likely to be important in *S. pyogenes* transmission via fomites (Gera & McIver, 2013; Trainor et al., 1999).

There remains a paucity of reports regarding the effect of environmental factors on *S. pyogenes*, despite the clinical significance of streptococcal diseases. The method used in this study may be applied to investigate a broader range of environmentally relevant temperatures and other factors affecting *S. pyogenes* viability outside of the human host. In future studies, the enumeration of colony counts at day 0 would provide the ideal baseline for measuring longitudinal survival, and it would be useful to understand the effect of different media (e.g., Amies vs. SMGG). Further, non‐culturable *S. pyogenes* may not necessarily be non‐viable and future studies could include measures of both CFU and metabolic activity (Trainor et al., 1999). Molecular methods may also form part of point‐of‐care tests for *S. pyogenes* (Pickering et al., 2020) but cannot distinguish live from dead bacteria and therefore cannot respond to questions of viability in the same way that culturing can.

## CONCLUSION

The viability and successful culture of *S. pyogenes* cells from inoculated swabs stored in SMGG is affected by storage temperature such that freezing is preferable over refrigeration. Ultimately, further research is needed to understand the dynamics of *S. pyogenes* environmental survival and transmission. The methodology used in this study may therefore be practical for future studies.

## AUTHOR CONTRIBUTIONS

**Kate Summer:** Writing – original draft; writing – review and editing; visualization; formal analysis. **Steven Y. C. Tong:** Writing – review and editing; supervision; funding acquisition. **Jonathan R. Carapetis:** Writing – review and editing; supervision; funding acquisition. **Asha C. Bowen:** Writing – review and editing; investigation; conceptualization; methodology; funding acquisition.

## CONFLICT OF INTEREST STATEMENT

The authors declare no conflicts of interest.

## Supporting information


**Data S1.** Supporting Information.

## Data Availability

The data that supports the findings of this study are available as supplementary material and the manuscript.
